# N-acetylcysteine inhibits bacterial lipopeptide-mediated neutrophil transmigration through the choroid plexus in the developing brain

**DOI:** 10.1186/s40478-019-0877-1

**Published:** 2020-01-23

**Authors:** Amin Mottahedin, Sandrine Blondel, Joakim Ek, Anna-Lena Leverin, Pernilla Svedin, Henrik Hagberg, Carina Mallard, Jean-Francois Ghersi-Egea, Nathalie Strazielle

**Affiliations:** 1 Fluid Team, Lyon Neurosciences Research Center, INSERM U1028, CNRS UMR5292, University Claude Bernard Lyon-1, Lyon, France; 2 Department of Physiology, Institute of Neuroscience and Physiology, Sahlgrenska Academy, University of Gothenburg, Gothenburg, Sweden; 3 Blood-Brain Interfaces Exploratory Platform BIP, Lyon Neurosciences Research Center, Lyon, France; 4 Centre for the Developing Brain, Division of Imaging Sciences and Biomedical Engineering, King’s College London, King’s Health Partners, St. Thomas’ Hospital, London, UK; 5 Centre of Perinatal Medicine & Health, Department of Obstetrics and Gynecology, Sahlgrenska Academy, University of Gothenburg, Gothenburg, Sweden; 6 Brain-i, Lyon, France

**Keywords:** Blood-brain barriers, Perinatal injury, Toll-like receptor, Innate immune system, Neuroinflammation, Neuroprotection

## Abstract

The etiology of neurological impairments associated with prematurity and other perinatal complications often involves an infectious or pro-inflammatory component. The use of antioxidant molecules have proved useful to protect the neonatal brain from injury. The choroid plexuses-CSF system shapes the central nervous system response to inflammation at the adult stage, but little is known on the neuroimmune interactions that take place at the choroidal blood-CSF barrier during development. We previously described that peripheral administration to neonatal mice of the TLR2 ligand PAM3CSK4 (P3C), a prototypic Gram-positive bacterial lipopeptide, induces the migration of innate immune cells to the CSF. Here we showed in neonatal rats exposed to P3C that the migration of neutrophils into the CSF, which occurred through the choroid plexuses, is abolished following administration of the antioxidant drug N-acetylcysteine. Combining light sheet microscopy imaging of choroid plexus, a differentiated model of the blood-CSF barrier, and multiplex cytokine assays, we showed that the choroidal epithelium responds to the bacterial insult by a specific pattern of cytokine secretion, leading to a selective accumulation of neutrophils in the choroid plexus and to their trafficking into CSF. N-acetylcysteine acted by blocking neutrophil migration across both the endothelium of choroidal stromal vessels and the epithelium forming the blood-CSF barrier, without interfering with neutrophil blood count, neutrophil tropism for choroid plexus, and choroidal chemokine-driven chemotaxis. N-acetylcysteine reduced the injury induced by hypoxia-ischemia in P3C-sensitized neonatal rats. Overall, the data show that a double endothelial and epithelial check point controls the transchoroidal migration of neutrophils into the developing brain. They also point to the efficacy of N-acetylcysteine in reducing the deleterious effects of inflammation-associated perinatal injuries by a previously undescribed mechanism, i.e. the inhibition of innate immune cell migration across the choroid plexuses, without interfering with the systemic inflammatory response to infection.

## Introduction

Prematurity and other perinatal complications including early-onset systemic infections, neonatal meningitis, or hypoxic/ischemic episodes can induce severe neurological deficiencies including cerebral palsy and are associated with an increased risk of neuropsychiatric sequelae such as autistic and schizophrenic syndromes in later life. The etiology of neurological impairments that are linked to perinatal injuries is likely multifactorial [21], and often involves an infectious or pro-inflammatory component [11, 21, 23, 61].

The neuroimmune interactions involved in the response of the central nervous system (CNS) to inflammation are tightly regulated. The CNS is protected against the invasion of immune cells by cellular barriers which comprise the cerebrovascular walls and the choroid plexuses (ChPs). Under physiological conditions, selected blood-borne immune cells pass the ChP barrier in small numbers and patrol the cerebrospinal fluid (CSF)-filled compartments performing CNS immunosurveillance [19, 46]. Owing to their early differentiation and maturation, the ChPs represent an important blood-brain interface for the developing brain [19]. Little is known however on the neuroimmune interactions that take place at the choroidal blood-CSF barrier during development. The CSF becomes pro-inflammatory in different perinatal pathologies, with elevated concentrations of chemokines and cytokines [6, 23, 44], and/or elevated numbers of monocytes and polymorphonuclear neutrophils (PMN). These cells are also found in CSF during meningitis [24], which is a leading cause of death among neonates [32], and a risk factor for neurological syndromes such as cerebral palsy [12, 31]. Increased immune cells are also observed in the CSF of newborns suffering from a peripheral infection (e.g. urinary track infection) [10, 54, 65].

The Toll-like receptors (TLRs), forming one family of pattern-recognition receptors, sense and respond to various stimuli originating from microbial pathogens or injured cells [2]. TLR2 has a pivotal role in inflammatory responses to Gram-positive bacteria and mycoplasma [57] which are responsible for a large proportion of perinatal infections such as chorioamnionitis and neonatal sepsis [16, 48, 53, 58]. We previously reported that peripheral TLR2 activation sensitizes mice to hypoxic-ischemic brain injury [38], that repeated stimulation of TLR2 impairs brain development in neonatal mice [13], and that *Staphylococcus epidermidis* induces brain injury in neonatal mice, partly via a TLR2-dependent pathway [5]. Collectively, these data indicate that the consequences of a transient bacteremia in early life may be more severe than commonly appreciated, even without CNS invasion by the pathogens. Systemic activation of TLR2 by peripheral exposure to the lipopeptide PAM3CSK4 (P3C) leads to a substantial infiltration of leukocytes, mainly neutrophils and monocytes, in the CSF and brain of neonatal mice [37]. The concurrent accumulation of these leukocyte subtypes in the choroidal tissue suggests that ChPs are a major site of immune cell trafficking into the CSF [36, 37, 52]. Neutrophils produce large amounts of reactive oxygen species which play a central role in the inflammatory response [34]. We and others previously showed that the antioxidant therapeutical compound N-acetylcysteine (NAC) protects the neonatal brain from injury in animals subjected to lipopolysaccharide exposure and hypoxia-ischemia [33, 62]. This drug also improves the glutathione-dependent antioxidant machinery at the ChPs [20]. The mechanisms by which NAC counteracts neuroinflammation in the context of perinatal injuries remain however poorly understood. Whether NAC treatment can influence the neuroinflammatory response by reducing immune cell invasion into the CSF has not been investigated.

We tested the hypotheses that peripheral exposure to the bacterial lipopeptide P3C induces innate immune cell migration across the ChPs in neonatal rats, and that NAC blocks this choroidal transmigration. The endothelium of the choroidal vessels is fenestrated and permeable, allowing blood-borne compounds to readily access the choroidal stroma. The actual barrier to soluble molecules between blood and CSF lies in the choroidal epithelium whose neighboring cells are joined by tight junctions. Immune cell trafficking from blood to CSF through the ChPs is more complex as it implies migration across both the endothelial and the epithelial cellular interfaces. Combining in vivo CSF sampling in neonatal rats, light sheet microscopy analysis of ChPs and a cellular model of the blood-CSF barrier, we showed that neutrophils cross the choroidal interface upon peripheral exposure to P3C. We demonstrated that NAC does not modify the tropism of neutrophils for the ChP, but blocks their migration across both the endothelial and the epithelial barriers, in a chemotaxis independent manner, preventing them to reach the CSF. Finally we showed that NAC-mediated blockage of PMN trafficking across the ChP into the CSF reduces the injury observed following hypoxia-ischemia in P3C-sensitized newborn rats.

## Materials and methods

### Animals

Female Sprague-Dawley or Wistar rats with their litters were purchased from Janvier (Le Genest Saint Isle, France), and kept under a 12 h light/dark cycle with ad libitum access to food and water. All animal procedures were conducted in accordance with guidelines of the French Ethical Committee, the European Union and Swedish Board of Agriculture. They were approved by the local ethical committee for animal experimentation CEEA-55 and the Gothenburg Animal Ethics Committee (Dnr 1–2016 to Henrik Hagberg).

### Rat treatment, CSF and blood collection, leukocyte count

Eight-day-old (P8) rats were injected intraperitoneally (i.p.) with 1 or 5 mg/kg P3C (Invivogen, France), 0.3 mg/kg ultrapure LPS (List Biological Laboratory, USA), or 0.9% saline. The dosages were chosen based on our previous work in mice [37]. In some experiments, N-acetylcysteine (Sigma-Aldrich) was injected i.p. together with P3C or saline at the dosage of 200 mg/kg, which is in the range of pharmacologically active doses in hypoxia-ischemia [62]. Fourteen hours after injection, rats were sacrificed with pentobarbital. The CSF was collected from the cisterna magna using a glass capillary. In some animals, blood was also collected by cardiac puncture. Leukocytes and PMNs were counted in a Bürker chamber after staining with Türk’s solution (Sigma-Aldrich).

### Immunophenotyping of leukocyte subsets in CSF

CSF samples (5 μl) were spotted on Superfrost PLUS glass slides (Thermo Scientific), dried at 37 °C, and fixed in − 20 °C acetone/methanol solution (1/1) for 2 min. Immunostaining was performed as described previously [45]. Primary antibodies were polyclonal rabbit anti-human myeloperoxidase (MPO, Dako, 1 μg/ml) and mouse anti-rat CD68 (Serotec, 1.67 μg/ml) to stain neutrophils and monocytes respectively, or mouse anti-rat CD45RA (BD Pharmingen, 2.5 μg/ml) and rabbit anti-rat CD3 (Abcam, 0.5 μg/ml) to stain B and T cells respectively. Fluorescent secondary antibodies from Invitrogen were used at 2 μg/ml. Nuclei were stained with 0.1 μg/ml DAPI (Roche Diagnostics) for 10 min. Images were acquired with a Zeiss Axio Imager.Z1 fluorescence microscope and analyzed using the Axiovision software 4.7.

### Plasma concentration-time curves and in vivo blood-CSF barrier permeability measurement

Plasma concentration-time curves were determined and blood-CSF barrier permeability measured as described previously [17]. Briefly, [^14^C]-sucrose, [^14^C]-urea, [^14^C]-caffeine or NAC were injected i.p. Plasma concentration-time curves were obtained by collecting blood between 3 and 30 min after injection, and up to 6 h for NAC. In some animals, blood sampling was followed immediately by CSF sampling through the cisterna magna. Average CSF sampling times were 20 min for sucrose, 5 min for caffeine, 12 min for urea, and 22 min for NAC. The CSF influx constant was calculated as:

$$ {K}_{in\kern0.30em CSF}={C}_t/ AU{C}_{0\to t} $$

where C_t_ is the labeled compound concentration in the CSF at the time of sampling t, and AUC_0 → t_ is the area-under-the-curve from time 0 to t recalculated from the concentration-time curve and from the plasma concentration measured immediately before CSF sampling. Radiolabeled compounds were analyzed by liquid scintillation [17], and NAC concentrations were measured by high performance liquid chromatography.

### Imaging of whole choroid plexus by selective plane illumination microscopy

Choroid plexuses were dissected under a binocular microscope, and fixed in 4% paraformaldehyde. Reca-1 (mouse antibody, AbD Serotec) and MPO immunostaining was performed as described [30, 45]. Tissues were imaged by selective plane illumination microscopy (SPIM), using a Lightsheet Z.1 microscope equipped with a sCMOS PCO camera (Zeiss). Images were deconvolved using Huygens 17.10 software (SVI) and analyzed with Imaris 7.2 (Bitplane) following 3D reconstruction. The vascular volume was measured based on Reca-1 staining. Myeloperoxidase-positive cells were counted in four to eight portions of each ChP viewed under rotation as illustrated in Additional file 1. Viewing under variable angles permitted to discriminate cells within the choroidal vascular space from those extravasated in the stroma. For each portion, results were normalized to the vascular volume measured in the tissue volume of interest, and values obtained in the different portions were averaged. In preliminary experiments comparing the perfused and non-perfused animals for SPIM analysis, we observed no difference in the total number of CD45-positive or MPO-positive cells in the choroidal tissue (data not shown). In non-perfused samples, the non-adherent leukocytes that were circulating in the choroidal vessels at the time of sacrifice were not fixed and were likely lost during the various incubations and washes of the immunostaining process.

### Choroid plexus epithelial cell culture

Primary cultures of choroid plexus epithelial cells (CPECs) were prepared and cultured as described [50, 51]. Cell monolayers used to measure chemokine secretion and to produce conditioned medium were cultured on the upper face of Transwell Clear inserts (6.5 mm diameter, 0.33 cm^2^ surface, 0.4 μm pore size, Corning B.V. Life Sciences, Amsterdam, The Netherlands). Cell monolayers used for transepithelial migration studies were cultured on the lower face of Transwell Clear inserts (6.5 mm diameter, 0.33 cm^2^ surface, 3.0 μm pore size), [50]. Experiments were performed 5 days after confluence. Cell-free laminin-coated inserts used for chemotaxis studies were kept in the same conditions. The paracellular integrity of cell monolayers was evaluated by sucrose permeability measurement as described [51].

### Leukocyte transmigration assay

Prior to the transmigration assay, CPEC monolayers were treated for 14 h with saline, 1 μg/ml P3C or P3C + 25 μM NAC added in the basolateral chamber. P8 rats were injected i.p. with 1 mg/kg P3C, and blood was collected 14 h later. Red blood cells were removed by dextran sedimentation and hypotonic lysis as follows: blood was mixed with 0.5 volume of 6% dextran solution in 0.15 M NaCl, and left unstirred for 45 min at room temperature. Leukocytes were collected from the top layer by centrifugation and resuspended in ice cold water for 30 s. Isotonicity was restored with ice-cold 0.6 M KCl. Cells were pelleted and resuspended in CPEC culture medium. Leukocytes were counted using Türk’s solution, and added to the basolateral side of CPECs at a concentration of 3 × 10^5^ cells per filter. Five hours later, the culture medium of the opposite chamber was collected and centrifuged at 500 *g* for 10 min. The transmigrated cells were resuspended in *ca* 50 μl medium. This suspension was used both for leukocyte counting and immunophenotyping as described above for CSF leukocytes.

### Chemotaxis assay

CPECs were treated with either P3C or P3C + NAC for 14 h as above. The conditioned apical medium was collected, centrifuged at 120 *g*, and either used fresh or kept at − 80 °C until used for chemotaxis studies. Neutrophils were isolated from P8 rats that were treated for 14 h with P3C. Blood was loaded on Ficoll Premium 1077 (GE Healthcare), and centrifuged at 400 *g* for 45 min. Neutrophils were collected at the surface of the pellet, and contaminating red blood cells were removed as described above. Isolated PMNs were resuspended in culture medium, added in the upper chamber of cell-free laminin-coated filters (3 x 10^5^ cells), and allowed to migrate towards CPEC-conditioned medium in the bottom chamber. Migrating cells were collected after 90 min and treated as above for counting.

### Multiplex cytokine assay

P8 rats were injected with P3C, P3C + NAC or saline as described above. Fourteen hours later, rats were sacrificed and plasma and CSF were collected. CPECs were treated with P3C, P3C + NAC or saline at their basolateral side and media were collected from both apical and basolateral compartments 14 h later. Before treatment, the culture media was replaced with F12/DMEM + 0.1% BSA to be compatible with the cytokine assay. Bio-Plex Pro™ Rat Cytokine 24-plex cytokine assay was performed following manufacturer instructions. CSF and plasma samples were diluted 1:5 in diluent buffer and the media from treated epithelial cells was diluted 1:2 in untreated media. The cytokines were measured on a Bio-Plex 200 Systems (Bio-Rad). Data were obtained and presented per ml for CSF and plasma data or per filter for CPEC secretion data. For the analytes that were below the detection limit, a value was set as half of the detection limit.

### Hypoxic-ischemic brain injury

P8 rats were injected with P3C + saline or P3C + NAC as described above. Hypoxia-ischemia was performed as previously described [38]. Briefly, pups were anesthetized with isoflurane and the left carotid artery permanently ligated. They were returned to the dam for recovery for 1 h and then exposed for 50 min to hypoxia (10% O_2_), which results in unilateral cerebral hypoxia-ischemia. Pups were sacrificed after one week with an overdose of thiopental sodium, and perfused with saline followed by 5% paraformaldehyde (Histofix, Histolab, Sweden). Brains were sampled post-fixed overnight in Histofix, dehydrated, embedded in paraffin and serially cut in 10-μm thick coronal sections. Three evenly spaced sections (L1-L3) at 500-μm intervals were taken through the hippocampus (levels corresponding to − 2.40 to − 3.40 of Bregma according to the stereotaxic coordinates of a P14 rat brain [29]), and were analyzed for brain injury. In this model, the injury affects mostly hippocampus, cortex, and striatum. The hippocampus region was selected, as we previously showed that tissue loss in one representative brain section at hippocampal level was positively correlated to the total volume of brain tissue loss [3].

### Immunohistochemistry and brain injury analysis

Immunohistochemistry to visualize neurons and measure neuronal loss was performed as described previously [38]. Briefly, sections were boiled for 10 min in citrate buffer for antigen recovery and endogenous peroxidase activity was blocked by incubating sections in 3% H_2_O_2_ for 10 min. Non-specific binding sites were blocked with 4% horse serum and 3% bovine serum albumin in PBS for 1 h at room temperature. Sections were incubated with the primary antibody against microtubule-associated protein 2 (MAP-2; HM-2, 1:1000; Sigma-Aldrich) at 4 °C overnight followed by a 1 h-incubation at room temperature with a biotinylated secondary antibody. Vectastain ABC Elite kit was used to enhance the peroxidase activity following the manufacturer’s protocol. Sections were imaged using an Olympus BX60 microscope equipped with an Olympus DP72 camera, and Olympus cellSens v.1.18 software was used to capture the images. The immunopositive surface area was measured in both hemispheres using ImageJ. Neuronal loss percentage was calculated by subtracting the stained area in the injured hemisphere from the stained area in the uninjured hemisphere and then dividing the result by the stained area in the uninjured hemisphere.

### Gene expression analysis by quantitative real-time polymerase chain reaction

Total RNA was extracted from CPECs using the RNeasy Micro kit (Qiagen, Valencia, CA) according to the manufacturer’s instructions. All samples were treated on column with DNase I as recommended by the manufacturer. RNA was quantified using a NanoDrop spectrophotometer (Thermo Scientific, Wilmington, DE). Total RNA (1 μg) was reverse transcribed using the iScript Reverse Transcription Supermix (Bio-Rad, Hercules, CA, USA). Quantitative real-time PCR (qRT-PCR) was performed using the LightCycler FastStart-DNA Master SYBR Green I kit in the LightCycler® 2.0 Instrument (Roche Diagnostics GmbH, Mannheim, Germany) as previously described [30]. Primers designed using NCBI Primer-BLAST were for TLR2: CAGCTGGAGAACTCTGACCC (forward) and CAAAGAGCCTGAAGTGGGAG (reverse), and for the reference gene (dolichyl-phosphate N-acetylglucosaminephosphotransferase 1, Dpagt1): GCCCTCGACACCGTATGCCC (forward) and TGTCACCAGCCGGAGGCTCT (reverse). Results were analysed using the LightCycler® Software 4.1. (Roche), using the respective amplification efficiencies of the target and the housekeeping genes determined from standard curves generated by nonlinear regression analysis of crossing points (Cp) measured over serial dilutions of a cDNA pool.

### Analysis of NAC in CSF and plasma by high performance liquid chromatography

Ten-microliter samples (plasma or CSF) were mixed with 100 μl of 2 mM 5,5′-dithiobis(2-nitrobenzoic acid) (Sigma) to produce a NAC disulfide derivative and incubated at 37 °C for 10 min. CSF samples were directly injected on an analytical column Phenomenex Luna C18 (3 μm, 150 mm × 4,6 mm). Plasma samples were subjected to precipitation by adding 400 μl acetonitrile. Following a 15-min incubation at room temperature, proteins were pelleted for 10 min at 14000 rpm, and the supernatant was concentrated in a speed vacuum concentrator (Savant). The remaining volume was measured before injection onto the column. Samples were separated using a Shimadzu apparatus composed of a CBM-20A controller, a LC-10AT pump, a SIL-10ADvp auto sampler system and a SPD-10Avp UV-Visible detector set at a wavelength of 324 nm. The analytical conditions were as follows: mobile phases with elution gradient in min (A:B): 0–8 (100:0); 10–19 (0–100); 21–26 (100–0), with A being a 10/7/83 (v/v/v) mixture of 100 mM KH_2_PO_4_ pH 6/methanol/water and B being a 10/30/60 (v/v/v) mixture of the same components; flow rate at 1 ml/min; injection volume of 20 μl. The retention times for NAC disulfide derivative and 2-nitro-5-thiobenzoate were 8.5 and 16 min, respectively.

### Statistical analysis

Prism V6.01 (GraphPad Software) was used to perform the statistical analyses. Data were analyzed by Student t-test or One-way ANOVA followed by Tukey’s post hoc tests and presented as mean ± SEM or mean ± SD. Brain injury results were analyzed by Mann-Whitney non-parametric test.

## Results

### Peripheral administration of P3C induces leukocyte accumulation in the choroid plexus and leukocyte infiltration in CSF

**Fig. 1:**
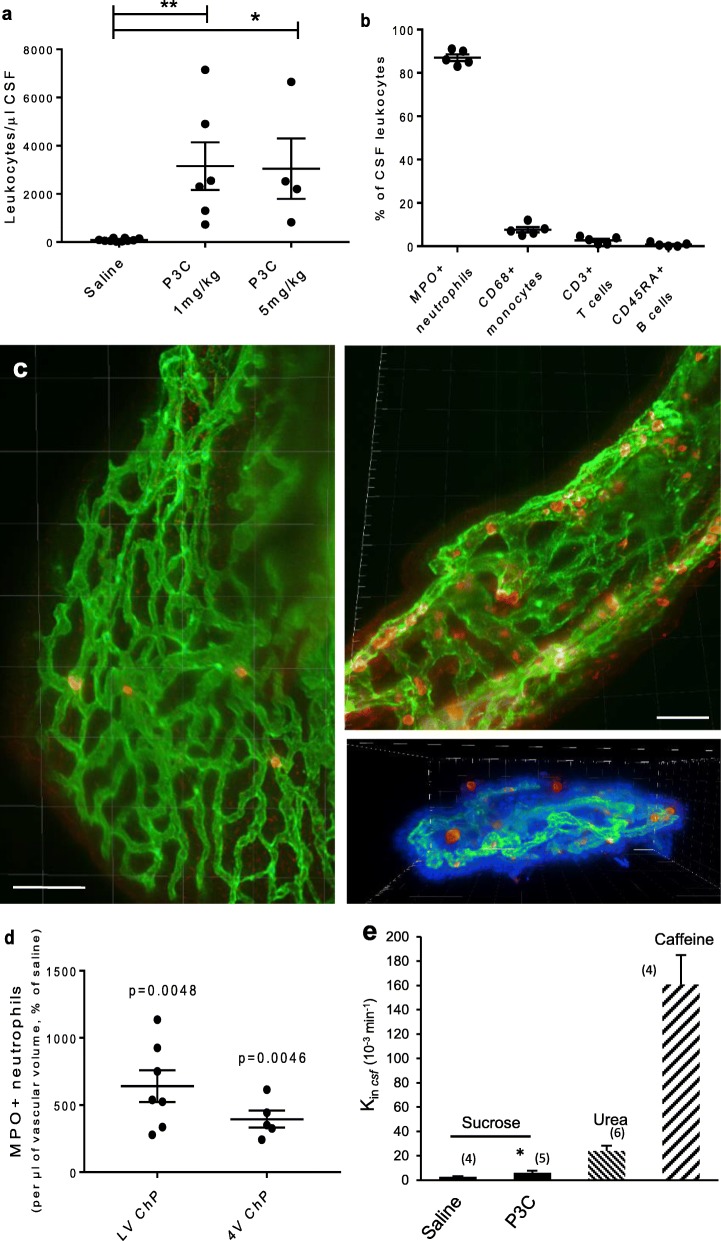
P3C induces a massive pleocytosis and a concomitant accumulation of neutrophils in choroid plexuses of neonatal rats. **a** CSF leukocyte counts after P3C treatment of P8 rats. Rats were injected i.p. with saline or P3C (either 1 or 5 mg/kg), and CSF leukocytes were counted 14 h later following Türk’s staining. Data from individual animals, and mean ± SEM are shown. * and**: statistically different from saline-treated animals, *p* < 0.05 and 0.01, respectively, one-way ANOVA followed by Dunnett’s multiple comparisons test. **b** Immunophenotypic analysis of CSF infiltrating leukocytes in P8 rats treated with 1 mg/kg P3C for 14 h. Neutrophils were identified as MPO^+^, monocytes as CD68^+^, T cells as CD3^+^ and B cells as CD45RA^+^ cells. Data from individual animals, and means ± SEM are shown. **c** Representative SPIM images of a portion of the ChP of the lateral ventricle from a saline-treated rat (left panel) and P3C-treated rat (right panels). MPO-immunopositive neutrophils in red, endothelial staining with Reca-1 in green. In the bottom right panel, nuclear staining with DAPI in blue allows to visualize the localization of some PMNs on the outer surface of the choroidal epithelium. **d** Quantification of MPO-immunopositive cells in ChPs of the lateral (LV ChP) and the fourth ventricle (4V ChP) from P3C-treated rats. Cell counts were normalized to the vascular volume of the region of interest. The data are expressed as % of cells counted in ChPs of saline-treated animals. These amounted to 13,009 ± 1884 and 15,092 ± 1347 cells per choroidal tissue volume containing 1 μl of vascular space in LV ChP and 4V ChP, respectively. Data from individual animals and mean ± SEM are shown. ** statistically different from saline-treated animals, *p* < 0.01, one-tailed Student t-test for unequal variance. **e** Blood-to-CSF permeability to sucrose in P8 rats following 14 h of treatment with saline or 1 mg/kg P3C. Permeability constants measured in untreated rats for urea (lowly permeable compound) and caffeine (highly permeable compound) are shown for comparison purposes. Data are presented as mean ± SD. * statistically different from saline-treated animals, *p* < 0.05, one-tailed Student t-test for unequal variance

Intraperitoneal administration of P3C to P8 rats induced a massive leukocyte infiltration into CSF at both 1 mg/ml and 5 mg/ml dosages, in comparison to saline injected animals (Fig. 1a). Similar to our previous experiments in mice, LPS injection did not lead to increased leukocyte migration to the CSF (48 ± 10 cells/μl; *p* > 0.05 compared to the saline condition). This underlines the specificity of the inflammatory response triggered by the Gram-positive lipopeptide versus a Gram-negative endotoxin. Immunophenotyping of leukocytes recovered in the CSF of P3C-treated rats identified a large majority of neutrophils (87 ± 1.5%), a few monocytes (7.6 ± 1.2%), and only a small proportion of T and B cells (Fig. 1b). We examined whether ChP could be a route of leukocyte entry accounting for this pleocytosis. Whole isolated ChPs were stained for neutrophils which represent the most abundant infiltrating cells, and analyzed by SPIM. In P3C-treated animals, we observed a substantial increase in the number of MPO-positive cells in the choroidal tissue from both the lateral and fourth ventricles (Fig. 1c showing the lateral ventricle ChP, and Fig. 1d for quantification in both ChPs). Occasional MPO-positive cells were found adhering on the apical membrane of the choroidal epithelium (Fig. 1c, lower right panel). These observations supported the hypothesis that the choroidal blood-CSF barrier is a major site of cell migration induced by P3C. The permeability of this barrier to sucrose was only slightly modified in response to P3C treatment (2-fold increase versus saline) (Fig. 1e). It remained however 4 times lower than the barrier permeability to urea measured in control animals. Urea is considered a low permeable compound, in contrast to the highly permeable caffeine ([49], and Fig. 1e). The mild effect on sucrose suggests that P3C-induced immune cell infiltration is a regulated process favoring an extensive early infiltration of neutrophils, which does not concur with a major alteration of the barrier tight junction integrity.

### Exposure to P3C induces neutrophil transmigration across choroid plexus epithelial cells

**Fig. 2:**
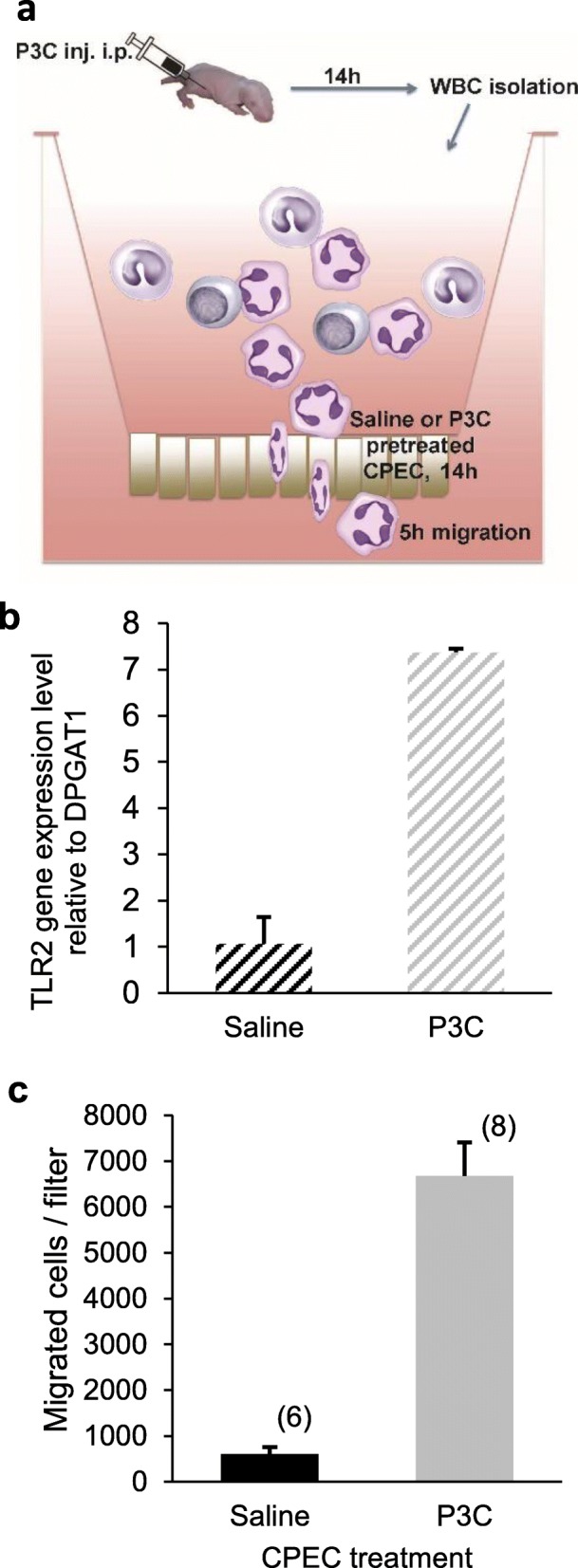
Exposure of the choroidal epithelium to P3C induces a selective migration of neutrophils across the blood-CSF barrier. **a** Schematic representation of the experimental setup for the transmigration assay. Choroid plexus epithelial cells are cultured on the lower side of microporous filters and are pretreated with P3C or saline. White blood cells isolated from P3C-treated animals are added to the stroma-mimicking basolateral medium in the upper chamber of the device and allowed to transmigrate for 5 h. Migrated cells are recovered from the CSF-mimicking apical medium in the lower chamber. **b** Effect of P3C on the expression level of TLR2 gene in CPECs. Cells were treated with saline or P3C for 14 h. Data are shown as mean ± SEM (*n* = 6 from two different CPEC cell preparations). **c** Effect of P3C on leukocyte migration across CPEC monolayers. Data are shown as mean ± SEM (n) from two different CPEC and white blood cell preparations

In order to probe into the mechanism of P3C-induced neutrophil infiltration via the choroidal route, we used an in vitro model of the blood-CSF barrier adapted for cell trafficking studies (Fig. 2a). The choroidal epithelial cells in culture expressed the gene for the P3C receptor TLR2 at basal levels that were similar to levels measured in ChPs isolated from untreated P9 rats (data not shown). Expression level was induced 7 times following treatment of CPECs with 1 μg/ml P3C for 14 h (Fig. 2b), recapitulating the increase observed in ChPs isolated from P3C-treated mice [52]. The lipopeptide did not intrinsically affect the choroidal barrier integrity. The permeability of CPECs to sucrose was 0.30 ± 0.02 and 0.31 ± 0.01 x 10^− 3^ cm.min^− 1^, respectively for control monolayers and monolayers treated with P3C for 8 h (mean ± SD, *n* = 4).

The transepithelial migration of peripheral white blood cells isolated from P3C-treated animals was examined across CPEC monolayers. Migration was substantially induced when the barrier cells were pretreated with P3C for 14 h compared to saline pretreatment (Fig. 2c). Immunophenotyping showed that MPO-positive PMNs represented 98 ± 0.8% (*n* = 8) of the transmigrated cells, while they accounted only for 55% of the leukocyte population initially presented at the basolateral side of the CPEC monolayers. The bacterial lipopeptide thus induced a distinct molecular signature on CPECs, favoring rapid and selective PMN trafficking across the choroidal epithelial barrier. Chemotaxis experiments performed with PMNs isolated from P3C-treated rats towards CPEC-conditioned medium sampled from the apical chamber provided evidence for the role of soluble factors secreted by the choroidal epithelium through its CSF-facing membrane. Migration of PMNs across cell-free filters was low in the presence of medium collected from quiescent CPECs and was stimulated 22 ± 4 times (mean ± SD, n = 4 from two independent assays) by medium conditioned by P3C-treated CPECs.

### N-acetylcysteine prevents P3C-induced pleocytosis in developing rats

**Fig. 3:**
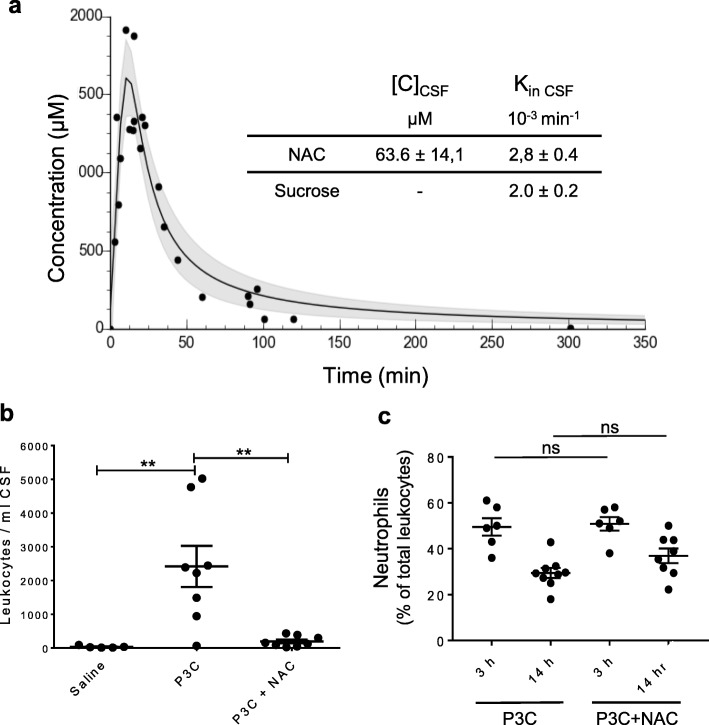
N-acetylcysteine prevents P3C-induced neutrophil infiltration into the CSF. **a** Blood and CSF bioavailability of NAC following intraperitoneal injection of 200 mg/kg NAC in P8 rats. For the plasma concentration-time curve of NAC, data were fitted using nonlinear regression analysis. Confidence band is shown in grey, for a confidence level set at 95%. The insert shows NAC concentration in CSF measured 22,6 ± 2.3 min after injection ([C]_CSF_) and the permeability constant K_in CSF_ calculated using the plasma concentration-time curve best fitted for the 0-to-60 min time frame. The K_in CSF_ value for sucrose is also indicated for comparison. **b** Effect of NAC on P3C-induced PMN infiltration in CSF. Rats were injected i.p. with saline, P3C or P3C + NAC, and CSF leukocytes were counted 14 h later following Türk’s staining. Data from individual animals, and mean ±+/− SEM are shown. **: *p* < 0.01, one-way ANOVA followed by Tukey’s multiple comparisons test. **c** NAC does not change PMN percentage within the total white blood cell population. Rats were injected i.p. with P3C or P3C + NAC, and white blood cells were isolated 3 and 14 h after treatment for myeloperoxidase immunostaining. No statistical difference in the percentage of neutrophils was found between P3C- and P3C + NAC-treated animals, at both time points. Data from individual animals and mean ± SEM are shown

Upon stimulation by bacterial components, neutrophils produce reactive oxygen species in large quan-tities as part of the innate response to infection [64]. We evaluated the effects of the common antioxidant NAC on P3C-induced leukocyte migration to the CSF. After intraperitoneal injection, NAC was rapidly resorbed with a maximal cardiac plasma concentration (1.6 mM) reached in 15 min (Fig.3a). The plasma concentration at that time was sufficient to drive NAC penetration into the CSF, although with a low permeability constant K_in CSF_, i.e. only slightly higher than that measured for sucrose (Fig. 3a, insert).

When co-administered with P3C, NAC almost fully inhibited leukocyte infiltration in CSF (Fig. 3 b). The percentage of PMNs in the leukocyte population was examined in blood sampled at 3 and 14 h after treatment. There was no difference between P3C- and P3C + NAC-treated animals at either time point (Fig. 3c), indicating that NAC blocking effect on cell trafficking to the CSF cannot be attributed to a depletion of PMNs in blood.

### N-acetylcysteine does not change neutrophil tropism toward the choroidal plexus but reduces their extravasation into the stroma

**Fig. 4:**
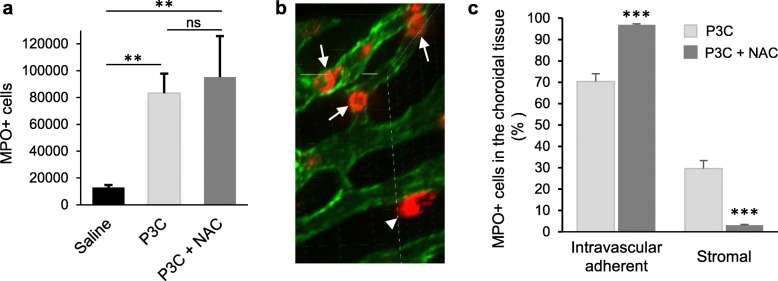
N-acetylcysteine blocks P3C-activated neutrophil extravasation into the choroidal stroma. **a** Effect of NAC on P3C-induced accumulation of PMNs in ChPs of the lateral ventricle. Rats were injected i.p. with saline, P3C or P3C + NAC, and ChPs were isolated 14 h later. Myeloperoxidase-immunopositive cells were quantified. Data shown as mean ± SEM (*n* = 4–8) represent the numbers of PMNs per choroidal tissue volume containing 1 μl of vascular space. ** statistically different from saline-treated animals, *p* < 0.01, one-way ANOVA followed by Tukey’s multiple comparison test. **b** Image of a portion of ChP viewed by SPIM under an angle that enables unequivocal identification of a stromal PMN (arrowhead) as compared to other intravascular (arrows) PMNs. Myeloperoxidase-immunopositive neutrophils in red, endothelial staining with Reca-1 in green. **c** Effect of NAC on the distribution of MPO-immunopositive cells between the intravascular and stromal spaces of ChPs isolated from the lateral ventricle. Data are shown as mean ± SEM (n = 4–8). *** statistically different from P3C-treated animals, *p* < 0.001, one-tailed Student’s t-test assuming unequal variance.

The analysis of whole ChPs isolated from P3C- and P3C + NAC-treated animals by SPIM showed that in contrast to its effect on pleocytosis, NAC did not modify the total number of PMNs associated with the choroidal interface (Fig. 4a for ChP of the lateral ventricle, not shown for ChP of the fourth ventricle). By viewing three-dimensional reconstructions of ChPs under variable angles, we were able to unequivocally assess the localization of each MPO-positive cell relative to the RECA-positive endothelial lining (Fig. 4b). The spatial repartition of the PMNs within the ChP of the lateral ventricle was significantly different between the two treatments. In P3C-treated animals, a majority (2/3) of these cells were intravascular, the remaining third being located in the stromal space, following extravasation across the choroidal endothelium (Fig. 4c). Treatment with NAC almost abolished the process, reducing the proportion of stromal cells from 30 to 3% of the total neutrophil population. Similar results were obtained for the ChP of the fourth ventricle (data not shown). In both P3C and P3C + NAC conditions, intravascular PMNs were adherent to the endothelial cell wall as their number was not different in ChP of rats perfused with saline before tissue sampling compared with non-perfused ChPs (see Materials and Methods section).

### N-acetylcysteine reduces P3C-induced transepithelial migration of neutrophils but does not change the chemoattractant effectors released by the choroidal epithelium

**Fig. 5:**
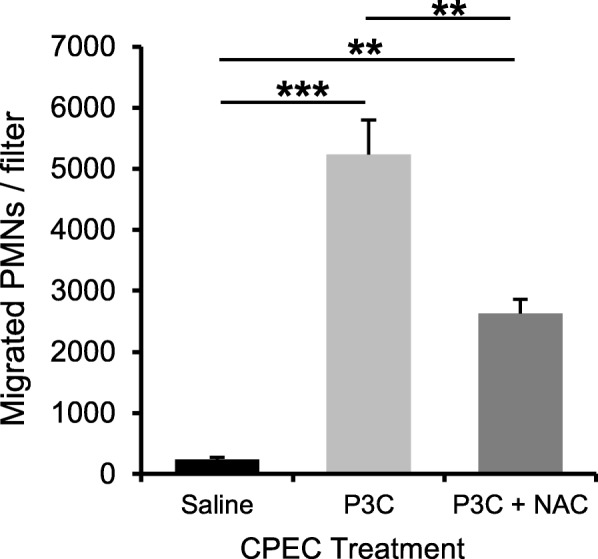
N-acetylcysteine decreases neutrophil transmigration across the choroidal epithelial barrier. Choroid plexus epithelial cells were pretreated with saline, P3C or P3C + NAC for 14 h. Total white blood cells were isolated from P8 rats treated with P3C for 14 h and added to the stroma-mimicking basolateral medium in the upper chamber. Migrated cells were recovered from the CSF-mimicking apical medium in the lower chamber after five hours, PMN were labeled and counted. Representative data are shown as mean ± SEM (n = 4). ** *p* < 0.01; ***p < 0.001, one-way ANOVA followed by Tukey’s multiple comparisons test. The experiment was repeated with different preparations of choroidal epithelial and white blood cells, and gave similar statistically significant differences

**Fig. 6:**
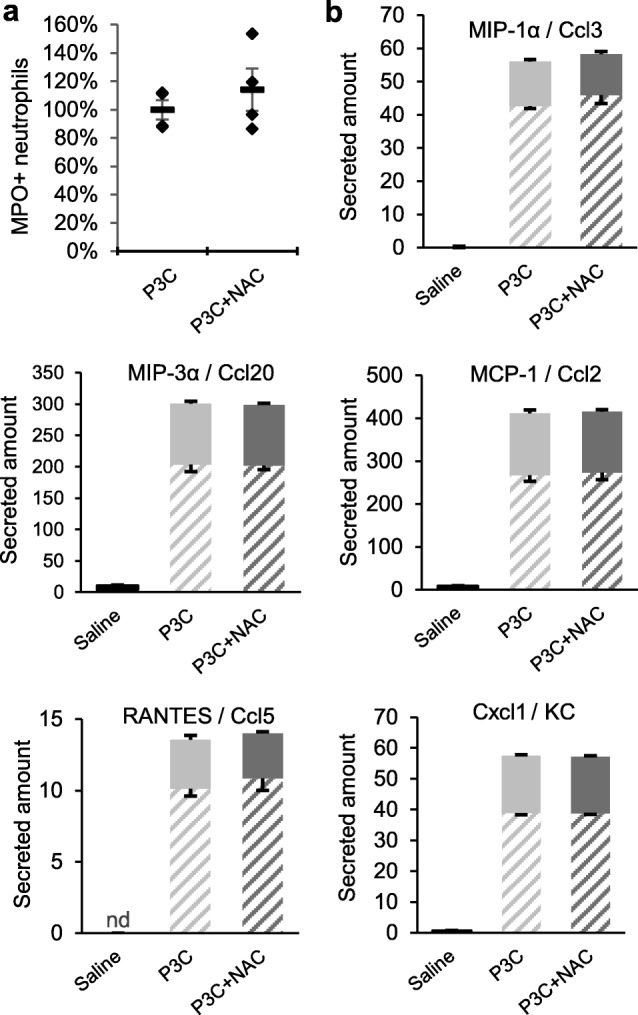
N-acetylcysteine does not change the chemoattracting activity of the choroidal epithelium towards neutrophils. **a** Chemotaxis of PMNs towards the CPEC-conditioned medium. CPECs were treated with P3C or P3C + NAC for 14 h and conditioned medium was recovered from the apical compartment. Neutrophils were isolated from P3C-treated rats, added in the upper chamber of cell-free filters and allowed to migrate towards CPEC-conditioned medium in the bottom chamber. Migrated cells were recovered and counted. Data from individual filters, and mean ± SEM are shown as percentages of the mean value calculated for P3C-treated cells. The difference between the 2 groups was not statistically significant, one-tailed Student t test for unequal variance. **b** Effect of P3C and NAC on chemokine secretion by CPECs. Choroidal epithelial cell monolayers were treated with saline, P3C or P3C + NAC for 14 h. Media were sampled from both apical and basolateral compartments. Data are expressed as ng per filter and are shown as mean ± SEM (*n* = 5). For all five chemokines, secreted amounts in the P3C and P3C + NAC groups were statistically different from the saline group for both apical (filled) and basolateral (hatched) mediums, *p* < 0.001, one-way ANOVA followed by Tukey’s multiple comparisons test. There was no difference between the P3C and P3C + NAC groups

To investigate whether NAC also influences leukocyte trafficking across the ChP by modulating molecular effectors on the epithelial barrier, we compared the migration of P3C-activated PMNs across CPEC monolayers pretreated with P3C or P3C + NAC. The drug concentration initially set at 25 μM decreased to 13 μM over the 14-h period in the incubation medium (data not shown). This dose approximated the concentration circulating in blood in NAC-treated P8 rats between 2 and 14 h after injection (Fig. 3a). The transepithelial migration of PMNs was largely reduced across NAC-pretreated CPECs (Fig. 5). Chemotaxis experiments comparing the activity of CPEC-conditioned medium towards P3C-activated PMNs showed that NAC does not modify the P3C-induced secretion of soluble chemoattractant factors by the choroidal epithelium (Fig. 6a). This was further confirmed by a more global analysis of the secretome of CPECs (Fig. 6b and Additional file 2). The secretion of five chemokines MCP-1/Ccl2, MIP-1α/Ccl3, RANTES/Ccl5, MIP-3α/Ccl20, and Cxcl1/KC was strongly increased in both apical and basolateral mediums of P3C-treated cells, consistent with the chemoattracting activity we reported. Amounts of these chemokines secreted by P3C + NAC-treated cells were not different (Fig. 6b). NAC had almost no effect either on the secretion of a large panel of cytokines induced by P3C (Additional file 2).

### N-acetylcysteine reduces cytokine and chemokine levels in CSF

**Fig. 7:**
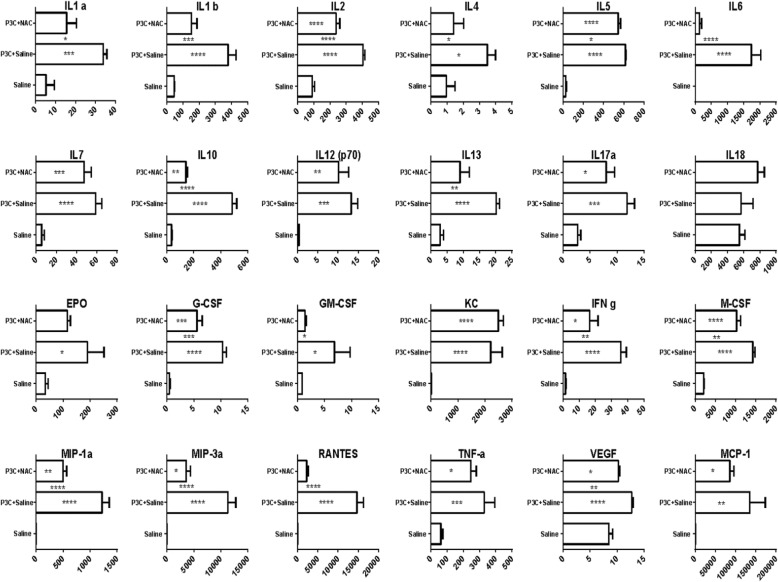
N-acetylcysteine decreases P3C-induced cytokine and chemokine release in CSF. P8 rats were injected i.p. with saline, P3C or P3C + NAC, and CSF was collected 14 h later. Cytokine concentrations measured by multiplex cytokine assay are presented in pg/ml as mean ± SEM (*n* = 7–9). * *p* < 0.05; ** *p* < 0.01; *** *p* < 0.001; **** *p* < 0.0001, one-way ANOVA followed by Tukey’s multiple comparisons test. Asterisks inside the bars show the comparison result between the treated and control group. Asterisks between bars show the comparison between P3C and P3C + NAC groups

The multiplex cytokine assay was also used to evaluate the effects of P3C on the neuroinflammatory status of CSF, and the influence of NAC treatment. P3C significantly increased the level of all measured cytokines except for IL18 compared to control group (Fig. 7). NAC treatment significantly diminished the amount of 16 of these cytokines in the CSF, including the three chemokines MIP-1α/Ccl3, MIP-3α/Ccl20, and RANTES/Ccl5, in comparison to P3C group (Fig. 7). In contrast, NAC did not modify the amounts of MCP-1/Ccl2 and KC/Cxcl1. The effect of NAC treatment on the levels of cytokines induced by P3C in plasma was different from the effect observed in CSF. Among 24 cytokines analyzed, P3C significantly increased the amount of 9 of them in the plasma in comparison to saline group while it decreased the level of IL18 (Additional file 3). Surprisingly, NAC treatment only decreased the level of RANTES/Ccl5 and to some extent increased the amount of the four other chemokines and of four cytokines in plasma in comparison to the levels measured in P3C-treated pups. Collectively these plasma, choroidal and CSF data suggest that infiltrating PMN contribute for a large part to the cytokines circulating in the CSF of P3C-treated animals. The prevention of PMN infiltration by NAC therefore reduce the level of cytokines in the CSF.

### N-acetylcysteine ameliorates P3C-sensitized hypoxic-ischemic brain injury

**Fig. 8:**
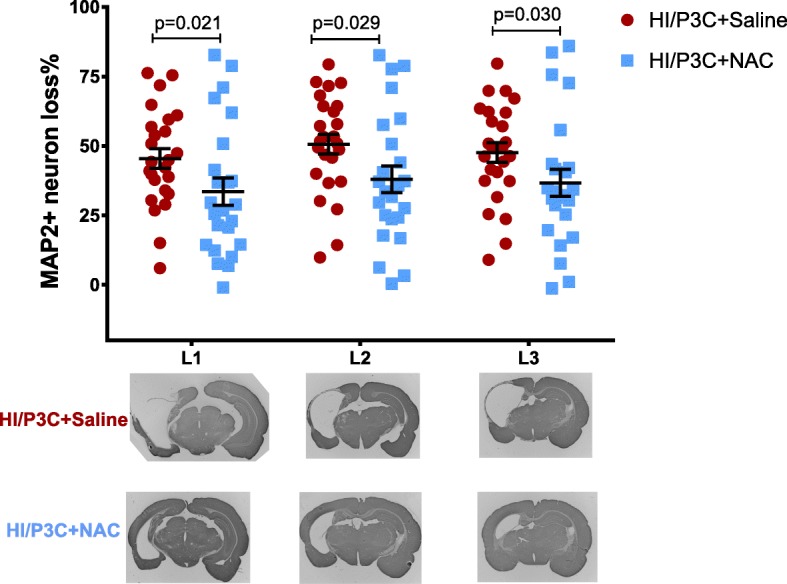
N-acetylcysteine reduces hypoxia-ischemia brain injury in P3C-sensitized neonatal rats. Neonatal rats were treated with P3C or P3C + NAC 14 h before being exposed to hypoxia-ischemia. One week later, mice were sacrificed and MAP 2-immunopositive area (neurons) was measured on three brain sections obtained at 3 different levels (represented in lower panels, Bregma − 3.4 to − 2.4). Results are expressed as a percentage of signal loss as compared to the contralateral uninjured hemisphere. Individual data and mean ± SEM are shown for the three levels L1, L2, and L3 (*n* = 25 and 24 for P3C- and P3C + NAC-treated rats, respectively). *P* values were calculated by Mann-Whitney non-parametric test

We and others previously showed that P3C sensitizes the developing brain to hypoxic-ischemic brain injury in mice and rats leading to an exacerbated pathological outcome [15, 38]. Therefore, we tested if NAC blocks the sensitizing effect of P3C and reduces the injury by co-injecting P3C and NAC 14 h before performing the hypoxia-ischemia procedure. As NAC concentration in blood had become insignificant (Fig. 2a), this timeline enabled to differentiate the effect of NAC on the P3C-mediated sensitization of the brain from the previously reported direct antioxidant effect of the drug on suffering neurons during the hypoxic-ischemic injury. The mean neuronal tissue loss was significantly reduced by 26, 25 and 22% at three hippocampal levels (Fig. 8) in pups injected with P3C + NAC compared to pups injected with P3C (Fig. 8).

## Discussion

The developing brain is especially susceptible to infection and inflammation of diverse viral and bacterial origins which can potentiate its vulnerability to hypoxic-ischemic insults or to other injuries [14, 23, 38]. In this study, we showed in line with our previous results generated in mouse [37], that peripheral exposure of developing rats to the bacterial lipopeptide P3C induces the recruitment of leukocytes to the ChPs and their infiltration into the CSF. The concurrent treatment of rats with the anti-oxidant drug NAC drastrically limited pleocytosis. It also reduced the sensitizing effect of P3C to hypoxic-ischemic brain injury. The SPIM technology enabled us to generate three-dimensional reconstructions of ChP and to precisely localize immune cells within the choroidal tissue. A differentiated and inverted cellular model of the blood-CSF barrier was used to assess changes in neutrophil transmigration across the choroidal epithelium. Combining these approaches, we were able to analyze both endothelial and epithelial steps of PMN trafficking across the ChP in order to understand this novel route of immune cell entry into the brain in the context of a neonatal bacterial infection and to approach the mechanism of action of the anti-oxidant drug.

In the healthy adult brain, ChPs participate to neuroimmune surveillance by allowing memory T cell entry into CSF spaces (reviewed in [19]). The ChPs are also involved in the trafficking of T cells in the inflamed adult brain [42], and specifically recruit anti-inflammatory M2 monocytes in a spinal cord injury model [46]. In traumatic brain injury, ChPs mediate the infiltration of neutrophils and monocytes to the distant cortical lesion site, the recruitment being orchestrated by chemokines secreted by the choroidal epithelial cells [55, 56]. In vitro, infection of a monolayer of porcine ChP epithelial cells by the Gram-positive bacteria *Streptococcus suis* induced an increase in neutrophil transmigration through these choroidal cells by a mechanism that is not fully understood [63]. We now showed that during the early postnatal period, the innate immune response to a systemic infectious stimulus, a Gram-positive lipopeptide, rapidly triggers the transmigration of neutrophils across the ChPs. Activation of TLR2 by the lipopeptide did not functionally alter the tightness of the blood-CSF barrier, but rather induced the secretion of different chemokines by ChP epithelial cells. Our chemotaxis data showed that these chemokines are required for neutrophil transmigration into the CSF. P3C-induced chemokine release occurred at both the apical and basolateral membranes. It is therefore possible that chemokines that are released into the choroidal stroma play a role in the tropism of blood circulating PMNs toward the ChPs, and in their extravasation.

The antioxidant drug NAC affected both the intrastromal PMN number as shown by SPIM analysis of whole ChPs and the transepithelial migration as analyzed using the in vitro blood-CSF barrier. This indicates that both the endothelium and the epithelium of ChPs play a role in setting the extent of PMN infiltration into the CSF following a peripheral infectious challenge. Past studies investigating cell migration across the blood-CSF barrier only concentrated on the epithelial layer, based on the fact that it displayed a tight phenotype and is the only barrier restricting molecular exchanges. Our data point at the endothelium, in addition to the epithelium, as a dynamic structure that needs to be taken into account when analyzing cell migration across the blood-CSF barrier.

NAC has been shown to ameliorate postnatal or later-life neurological outcome in different models of perinatal diseases, when administrated to the mother before birth or postnatally to the pups [1, 4, 7, 33, 62]. It has been the object of a positive clinical trial in the context of chorioamnionitis [26]. The mechanism involved in NAC action has however been little explored. According to the current hypothesis, relying mainly on in vitro studies and on experiments in adult model of brain injuries, NAC has an antioxidant effect on neural cells, especially glial cells, microglia and oligodendrocytes. This leads to cell protection, and for microglia, to an orientation toward an anti-inflammatory phenotype [1, 22, 47, 66]. Here we describe a novel mechanism of action by which NAC limits the development of a proinflammatory environment within the brain, by preventing activated innate immune cells from entering the CNS. Both PMN and choroid plexus cells may be targets of NAC. In the latter case, the high blood flow of ChPs relative to brain parenchyma in developing animals will favor NAC delivery to both endothelial and epithelial layers of ChPs [18]. NAC being negatively charged at physiological pH, is not expected to have a high cerebral bioavailability, which was confirmed by the low blood-CSF permeability that we measured in the neonatal animals (Fig. 3 insert). As NAC penetration into the CSF and brain is not required for its effect on neutrophil migration, the therapeutic efficacy in terms of neuroprotection does not suffer from the poor cerebral bioavailability of the drug.

The molecular events underlying the inhibiting effect of NAC on neutrophil trafficking across the ChP remain unknown. The tropism of neutrophils for ChPs and their adhesion to choroidal vessels did not change following NAC treatment. We showed in vivo and in vitro respectively, that the extravasation step and the subsequent migration step across the epithelium are both pharmacological targets of NAC. One limitation of the in vitro study is that NAC concentration in the medium does not match the concentration-time profile observed in vivo in plasma. We sought to minimize this limitation by exposing the cells to a low dose that approximated the average concentration circulating in blood prior CSF sampling. This concentration effectively blocked neutrophil trafficking across the barrier, as observed in vivo. Neutrophil migration is dependent on chemokine/cytokine secretion by the choroidal epithelium. Several studies showed that antioxidants can reduce the production of some cytokines by several types of cells exposed to inflammatory challenges [25, 41]. However NAC did not alter the pattern of chemokine secretion by the choroidal epithelium, while it decreased neutrophil transmigration (Figs. 5 and 6, and Additional file 2). The chemokine/cytokine upregulation observed in blood from P3C-treated animals was also not counteracted by NAC. This is in line with the absence of blood cytokine level changes in a clinical trial dedicated to the effect of NAC treatment on markers of inflammation in bipolar depression [40]. NAC is also unlikely to change the neutrophil migratory behavior in response to chemokines, as drug concentrations in the tenths to hundreds millimolar range were necessary before such effect was observed [28].

TLR2 activation induces the production of NADPH oxidase-derived reactive oxygen species including hydrogen peroxide and superoxide anion. Hydrogen peroxide has emerged as a chemotactic factor for neutrophils. It was shown in Zebra fish to promote the recruitment of leukocytes to the wounded site in both a paracrine and an autocrine manner [39]. Our transcriptomic analysis of ChPs [36] revealed that P3C specifically activates cytoskeleton remodeling pathways, including actin organization, which may be relevant to the transcellular or paracellular migration of leukocytes. Reactive oxygen species, particularly those derived from NADPH oxidases, are important second messengers in the regulation of the actin cytoskeleton [59]. Whether the canonical antioxidant activity of NAC blocks the hydrogen peroxide-mediated chemotaxis of neutrophils and/or impedes cytoskeleton rearrangement in the choroidal epithelial cells, and possibly also in neutrophils, require further investigations.

Modulating neuroinflammation is a promising neuroprotective strategy in infectious perinatal neurological diseases including meningitis [60]. It should also be valuable in perinatal diseases such as hypoxia-ischemia that are potentiated by a systemic infection sensitizing episode. NAC would be of particular interest in these injuries since according to our results it does not suppress the inflammatory response in the periphery which is necessary to control infections. We showed that NAC ameliorates TLR2-mediated CNS inflammation by inhibiting neutrophil infiltration. This pharmacological effect should be highly relevant as the vast majority of neonatal infections are caused by Gram-positive species [53]. We and other previously showed that systemic activation of TLR2 by P3C sensitizes the brain of neonatal mice and rats to subsequent hypoxic-ischemic injury [15, 38]. We here demonstrated that NAC ameliorates P3C-sensitized hypoxic-ischemic injury in the neonatal rat. NAC cerebral bioavailability is low (our data and [8]), and NAC treatment is ineffective in increasing antioxidant defense in the normal brain [8, 9, 27, 43]. During hypoxic/ischemic stress, NAC efficiently protects altered neurons by restoring reduced glutathione and cysteine levels or acting directly as an antioxidant molecule. These beneficial effects occur only when NAC is administered at the time of hypoxia [35, 62]. The short half-life measured for NAC in our single dose treatment likely precluded a direct action of the drug on brain cells during hypoxia-ischemia performed 14 h later. The protective effect of NAC on neuronal injury is therefore most likely due to its effect in reducing P3C-induced sensitization of the brain, rather than its antioxidant activity counteracting hypoxia-induced neuronal death. P3C-induced brain sensitization to hypoxia-ischemia involves neutrophil trafficking across the ChP, but other P3C-dependent sensitization mechanisms that could also be NAC-sensitive cannot be ruled out at that stage. Finally, NAC treatment of P3C-treated rats induced a decrease in the level of most cytokines and chemokines in CSF that paralleled the decrease in PMN pleocytosis. Infiltrating PMNs in P3C-treated animals may secrete to some extent these inflammatory mediators. Resident microglial cells activated by PMN-derived cytokines constitute another source for these factors which may contribute to recruit other leukocytes. Exceptions include the chemokines KC (Cxcl1) and MCP-1 (Ccl2), whose CSF levels did not decrease following NAC treatment. As these chemokines are secreted at equally high levels by CPECs exposed to P3C and P3C + NAC, the choroidal epithelium appears as a likely source of these two factors in CSF. Whether these choroidal chemokines are sufficient to trigger an inflammatory reaction in brain tissue despite the absence of infiltrating innate immune cells into the CSF remains to be established.

In conclusion, the present work showed that both the endothelial and the epithelial cells of the ChPs are check points for the migration of neutrophils into the developing brain that occurs following exposure to Gram-positive bacterial TLR2 activators. It also identified NAC as a drug candidate capable of preventing central inflammation, without interfering with the systemic inflammatory response to infection. NAC action occurs through a previously undescribed mechanism, i.e. the inhibition of innate immune cell migration across the choroid plexuses.

## Supplementary information

**Additional file 1.** Recruitement and extravasation of neutrophils in the choroid plexus of a neonatal rat following P3C treatment. Portions of choroid plexus in which MPO-positive neutrophils were stained in red, and the choroidal endothelium in green, were viewed by light sheet microscopy under rotation, to discriminate cells located within the choroidal vascular space from those which extravasated in the choroidal stroma. Only the horizontal rotation is shown here, but each MPO-positive cells was viewed from 3 different angles to ascertain the localization of the cell relative to the vasculature

**Additional file 2. **Levels of cytokines secreted by choroid plexus epithelial cells exposed to saline, P3C, or P3C + NAC for 14 h. CPECs were exposed to saline, P3C or P3C + NAC at their basolateral membrane. Apical and basolateral mediums were collected 14 h later and analyzed by multiplex cytokine assay. P3C increased the secretion of most cytokines, except for VEGF whose secretion was decreased. NAC had little effect on P3C-induced secretion. Values are expressed as pg per filter, mean ± SEM, *n* = 5. *, **, ***, statistically different from control, *p* < 0.05, 0.01 and 0.001, respectively. ^$^, P3C+NAC group statistically different from P3C group, *p* < 0.05, one-way ANOVA followed by Tukey’s multiple comparisons test

**Additional file 3. **N-acetylcysteine increases P3C-induced cytokine release in the plasma of neonatal rats. P8 rats were injected i.p. with saline, P3C or P3C + NAC, and plasma was collected 14 h later. NAC increased P3C-induced release of most cytokines in the plasma. Data in pg/ml are presented as mean ± SEM, *n* = 7–9. *p < 0.05; ** *p* < 0.01; ***p < 0.001; ****p < 0.0001, one-way ANOVA followed by Tukey’s post hoc test. Asterisks inside the bars show the statistical significance between treated and control groups. Asterisks between bars show the statistical significance between P3C and P3C + NAC groups

## Data Availability

All data generated or analysed during this study are included in this published article and its supplementary information files.

## Abbreviations

ChP: Choroid plexus
CNS: Central nervous system
CPEC: Choroid plexus epithelial cell
CSF: Cerebrospinal fluid
MPO: Myeloperoxidase
NAC: N-acetylcysteine
P3C: PAM3CSK4
PMN: Polymorphonuclear neutrophil
SPIM: Selective plane illumination microscopy
TLR: Toll-like receptor
