# Supplementary material for: N-acetylcysteine inhibits bacterial lipopeptide-mediated neutrophil transmigration through the choroid plexus in the developing brain
Source: Acta Neuropathol Commun. 2020 Jan 23;8:4. doi: 10.1186/s40478-019-0877-1 (PMC6979079; doi:10.1186/s40478-019-0877-1)
Supplement: Supplementary file 2 — Additional file 2. Levels of cytokines secreted by choroid plexus epithelial cells exposed to saline, P3C, or P3C + NAC for 14 h. CPECs were exposed to saline, P3C or P3C + NAC at their basolateral membrane. Apical and basolateral mediums were collected 14 h later and analyzed by multiplex cytokine assay. P3C increased the secretion of most cytokines, except for VEGF whose secretion was decreased. NAC had little effect on P3C-induced secretion. Values are expressed as pg per filter, mean ± SEM, n = 5. *, **, ***, statistically different from control, p < 0.05, 0.01 and 0.001, respectively. $, P3C+NAC group statistically different from P3C group, p < 0.05, one-way ANOVA followed by Tukey’s multiple comparisons test [file 40478_2019_877_MOESM2_ESM.pdf]

N-acetylcysteine inhibits bacterial lipopeptide-mediated neutrophil transmigration through the choroid plexus in the developing brain
Acta Neuropathologica Communications
Amin Mottahedin[1,2], Sandrine Blondel[3], Joakim Ek[2], Anna-Lena Leverin[2], Pernilla Svedin[2], Henrik Hagberg[4,5], Carina Mallard[2], Jean-Francois Ghersi-Egea[1,3], Nathalie Strazielle[1,6]
Corresponding author : N Strazielle, Brain-*i* and CRNL, Lyon, France, ns.brain.i@gmail.com

Additional file 2. Levels of cytokines secreted by choroid plexus epithelial cells exposed to saline, P3C, or P3C + NAC for 14 hours

Choroid plexus epithelial cells were exposed to saline, P3C or P3C + NAC at their basolateral membrane. Apical and basolateral mediums were collected 14 hrs later and analyzed by multiplex cytokine assay. P3C increased the secretion of most cytokines, except for VEGF whose secretion was decreased. NAC had little effect on P3C-induced secretion. Values are expressed as pg per filter, mean ± SEM, n=5. *, **, ***, statistically different from control, p<0.05, 0.01 and 0.001, respectively. $, P3C-NAC group statistically different from P3C group, p<0.05. one-way ANOVA followed by Tukey's multiple comparison test.

	**Saline**		**P3C**		**P3C-NAC**	
	**Basolateral**	**Apical**	**Basolateral**	**Apical**	**Basolateral**	**Apical**
**IL-1alpha**	117 ± 50	0 ± 0	445 ± 27***	114 ± 24**	398 ± 37***	104 ± 18**
**IL-1beta**	221 ± 82	0 ± 0	663 ± 75*	169 ± 28***	656 ± 122*	173 ± 29***
**IL-2**	1186 ± 56	0 ± 0	2004 ± 159**	543 ± 120**	2538 ± 166***$	592 ± 100**
**IL-4**	196 ± 7	71 ± 9	245 ± 9	55 ± 8	224 ± 14	56 ± 4
**IL-5**	265 ± 16	248 ± 47	1183 ± 33***	501 ± 15***	1036 ± 36***$	498 ± 15***
**IL-6**	0 ± 0	0 ± 0	9099 ± 842***	2707 ± 320***	8083 ± 795***	2850 ± 443***
**IL-7**	0 ± 0	0 ± 0	1370 ± 48***	473 ± 34***	1381 ± 107***	399 ± 45***
**IL-10**	9611 ± 1936	13721 ± 3259	494545 ± 22929***	157240 ± 6286***	474769 ± 5463***	138948 ± 6206***
**IL-12(p70)**	337 ± 92	0 ± 0	844 ± 84**	215 ± 52**	685 ± 45*	265 ± 26***
**IL-13**	47 ± 8	0 ± 0	86 ± 6**	16 ± 6	90 ± 8**	9 ± 5
**IL-17A**	0 ± 0	0 ± 0	62 ± 17**	43 ± 6***	82 ± 11***	42 ± 8***
**IL-18**	0 ± 0	0 ± 0	150 ± 45	115 ± 57	340 ± 101**	78 ± 49
**EPO**	62 ± 62	0 ± 0	652 ± 95***	185 ± 91	502 ± 86**	149 ± 44
**G-CSF**	0 ± 0	0 ± 0	380 ± 96*	44 ± 29	459 ± 96**	96 ± 45
**GM-CSF**	0 ± 0	0 ± 0	274 ± 32**	0 ± 0	192 ± 67*	0 ± 0
**IFN-gamma**	616 ± 56	142 ± 78	899 ± 50	120 ± 38	833 ± 106	227 ± 58
**M-CSF**	115 ± 23	108 ± 9	361 ± 29***	196 ± 11***	363 ± 27***	197 ± 7***
**TNF-alpha**	215 ± 99	168 ± 141	41948 ± 4512***	8355 ± 723***	37469 ± 3293***	7760 ± 641***
**VEGF**	176113 ± 3836	74913 ± 974	163509 ± 3042*	30347 ± 1031***	152141 ± 1380***$	30531 ± 1607***
