# Supplementary material for: N-acetylcysteine inhibits bacterial lipopeptide-mediated neutrophil transmigration through the choroid plexus in the developing brain
Source: Acta Neuropathol Commun. 2020 Jan 23;8:4. doi: 10.1186/s40478-019-0877-1 (PMC6979079; doi:10.1186/s40478-019-0877-1)

N-acetylcysteine inhibits bacterial lipopeptide-mediated neutrophil transmigration through the choroid plexus in the developing brain
Acta Neuropathologica Communications
Amin Mottahedin[1,2], Sandrine Blondel[3], Joakim Ek[2], Anna-Lena Leverin[2], Pernilla Svedin[2], Henrik Hagberg[4,5], Carina Mallard[2], Jean-Francois Ghersi-Egea[1,3], Nathalie Strazielle[1,6]
Corresponding author : N Strazielle, Brain-*i* and CRNL, Lyon, France
ns.brain.i@gmail.com

Additional file 3. N-acetylcysteine increases P3C-induced cytokine release in the plasma of neonate rats.

P8 rats were injected i.p. with saline, P3C or P3C+NAC, and plasma was collected 14 hours later. NAC increased P3C-induced release of most cytokines in the plasma. Data in pg/ml are presented as mean ± SEM, n=7-9. *p < 0.05; ** p< 0.01; ***p < 0.001; ****p< 0.0001, one-way ANOVA followed by Tukey's post hoc test. Asterisks inside the bars show the statistical significance between treated and control group. Asterisks between bars show the statistical significance between P3C and P3C+NAC groups.

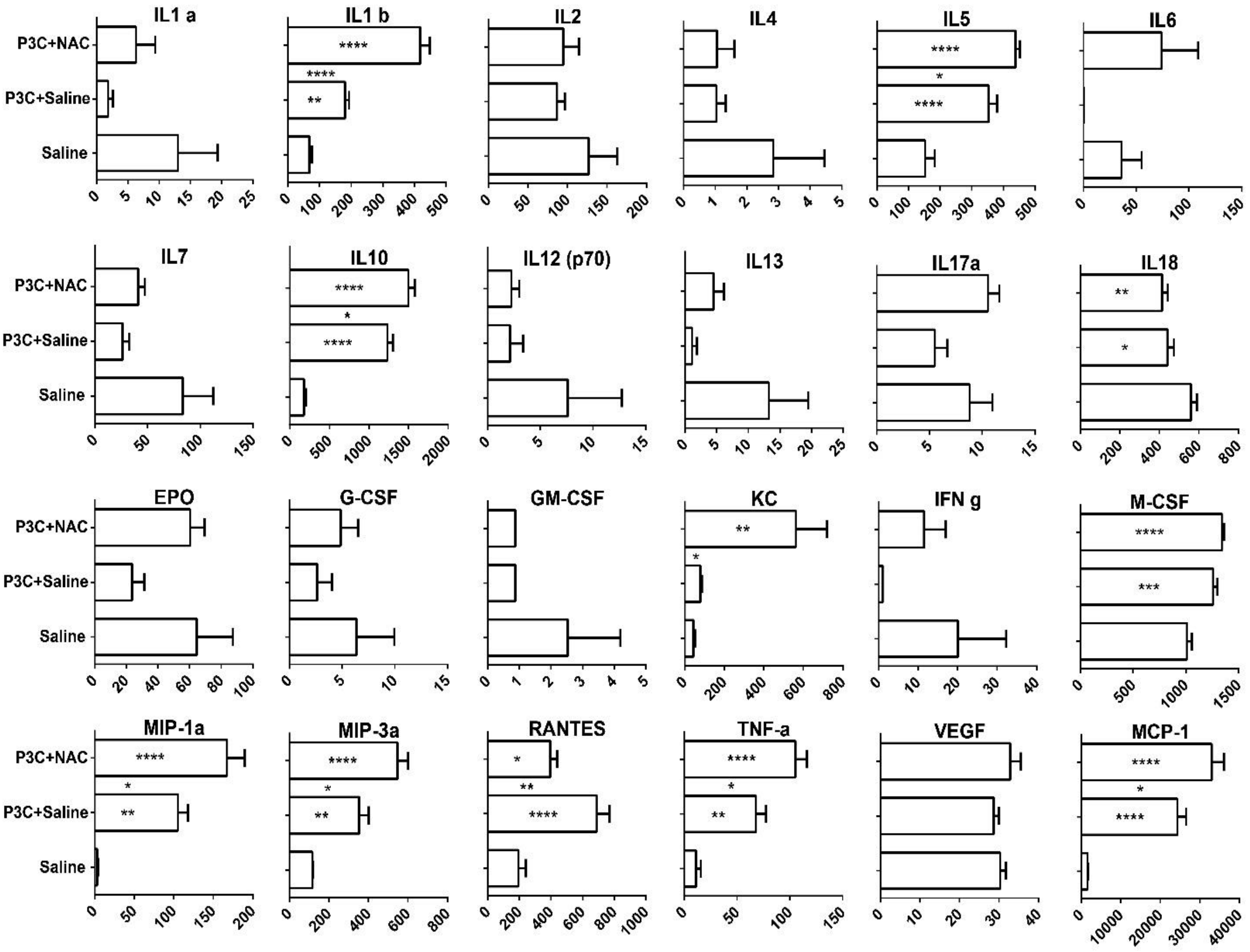

Supplement: Supplementary file 3 — Additional file 3. N-acetylcysteine increases P3C-induced cytokine release in the plasma of neonatal rats. P8 rats were injected i.p. with saline, P3C or P3C + NAC, and plasma was collected 14 h later. NAC increased P3C-induced release of most cytokines in the plasma. Data in pg/ml are presented as mean ± SEM, n = 7–9. *p < 0.05; ** p < 0.01; ***p < 0.001; ****p < 0.0001, one-way ANOVA followed by Tukey’s post hoc test. Asterisks inside the bars show the statistical significance between treated and control groups. Asterisks between bars show the statistical significance between P3C and P3C + NAC groups [file 40478_2019_877_MOESM3_ESM.pdf]
